# A New Imaging Platform for Visualizing Biological Effects of Non-Invasive Radiofrequency Electric-Field Cancer Hyperthermia

**DOI:** 10.1371/journal.pone.0136382

**Published:** 2015-08-26

**Authors:** Stuart J. Corr, Sabeel Shamsudeen, Leoncio A. Vergara, Jason Chak-Shing Ho, Matthew J. Ware, Vazrik Keshishian, Kenji Yokoi, David J. Savage, Ismail M. Meraz, Warna Kaluarachchi, Brandon T. Cisneros, Mustafa Raoof, Duy Trac Nguyen, Yingchun Zhang, Lon J. Wilson, Huw Summers, Paul Rees, Steven A. Curley, Rita E. Serda

**Affiliations:** 1 Department of Surgery, Division of Surgical Research, Baylor College of Medicine, Houston, TX, United States of America; 2 Department of Chemistry, Rice University, Houston, TX, United States of America; 3 Department of Surgical Oncology, University of Texas M.D. Anderson Cancer Center, Houston, TX, United States of America; 4 Department of Nanomedicine, Houston Methodist Research Institute, Houston, TX, United States of America; 5 Department of Biomedical Engineering, University of Houston, TX, United States of America; 6 Centre for Nanohealth, College of Engineering, Swansea University, Swansea, Wales, United Kingdom; 7 The Broad Institute, Cambridge, MA, United States of America; 8 Department of Mechanical Engineering and Materials Science, Rice University, Houston, TX, United States of America; Universidad Carlos III de Madrid; Instituto de Investigación Sanitaria Gregorio Marañon, SPAIN

## Abstract

Herein, we present a novel imaging platform to study the biological effects of non-invasive radiofrequency (RF) electric field cancer hyperthermia. This system allows for *real-time in vivo* intravital microscopy (IVM) imaging of radiofrequency-induced biological alterations such as changes in vessel structure and drug perfusion. Our results indicate that the IVM system is able to handle exposure to high-power electric-fields without inducing significant hardware damage or imaging artifacts. Furthermore, short durations of low-power (< 200 W) radiofrequency exposure increased transport and perfusion of fluorescent tracers into the tumors at temperatures below 41°C. Vessel deformations and blood coagulation were seen for tumor temperatures around 44°C. These results highlight the use of our integrated IVM-RF imaging platform as a powerful new tool to visualize the dynamics and interplay between radiofrequency energy and biological tissues, organs, and tumors.

## Introduction

Interactions of high-frequency radiowaves (13.56 MHz) with tissues and nanomaterials in biological tissues are currently being investigated as a therapeutic platform for non-invasive cancer hyperthermia therapy. The unique dielectric properties of cancerous tissues favor radiofrequency (RF) energy absorption and conversion to heat and is hypothesized to be further accelerated through the use of RF-energy absorbing nanomaterials such as gold nanoparticles and single-walled carbon nanotubes. Enhanced tumor heating is due to larger dielectric losses within tumor tissue compared to normal tissues[1] and has found applications in clinical hyperthermia[2]. Much work had been focused on measuring and interpreting the temperature distribution[3] and dielectric properties of various healthy and cancerous animal tissues across radio- and micro-wave frequencies[4–6]. Compared to other nano-based photothermal hyperthermia approaches RF therapy offers the advantage of greater tissue penetration depths (~5–30 cm), which is due to the relatively long RF wavelengths (~22 m at 13.56 MHz), when compared to the subsurface millimeter penetration depths of infrared (IR) and near-infrared (NIR) light.

In a bid to further increase differential-heating rates and RF-induced cancer cytotoxicity, several studies have shown the heating characteristics[7–12]; biological toxicity[13–20]; electrical interactions[11, 21–24]; and feasibility of nanomaterial interactions with RF energy and their use as a potential medical hyperthermia adjuvant. Despite the continuing evolution of the various roles that nanomaterials play in localized heat generation and cytotoxicity, both within simplified aqueous solutions and biological materials, much work has been achieved towards understanding the basic science behind RF interactions with biological tissues with the potential for synergy to exist with clinically approved chemotherapy agents such as abraxane, cetuximab, and gemcitabine [25]. The referenced review articles by Collins *et al*. and Liu *et al*. offer a comprehensive and concise overview of the field [26, 27].

A means of directly visualizing the interplay between biological tissues and RF electric-fields, enabling insight into the fundamental processes and basic science behind this therapy, has been missing. As of yet there has been no design for capturing these dynamic events, particularly due to the difficulty in integrating a high-power electric field generator into various imaging modalities. Herein, we present an integrative system combining RF exposure with high-resolution intravital microscopy (IVM) (RF-IVM) to allow *real-time in vivo* fluorescent imaging of RF-induced biological effects. IVM, using confocal and or multiphoton excitation technology, is a powerful technique for imaging live animals at high-resolution with the ability to reach tissue depths of several hundred micrometers. With this technique, investigators are able to evaluate tissue and cellular responses over time and in three-dimensional space in living tissue under natural physiological conditions [28]. The data presented in this study demonstrate that (i) a high-power RF generator system (200 W, ~15 kV/m) can successfully be retro-fitted into a Nikon A1R IVM system without hardware damage or imaging artifacts; and (ii) the integrated IVM-RF system allows the imaging of mild hyperthermia-induced dynamic events (< 41°C), such as increased tumor perfusion of systemically administered fluorescent tracers (albumin and FITC-dextran) as well as vessel deformation and coagulation observed across the temperature range 44–49°C. Given these results, we anticipate that the IVM-RF system will enable us to image RF-induced biological events such as changes in vascular permeability, alterations in tissue integrity, influence on nanoparticle and drug accumulation, tissue penetration, and cellular migration events.

## Materials and Methods

### Portable-RF system

**Fig 1:**
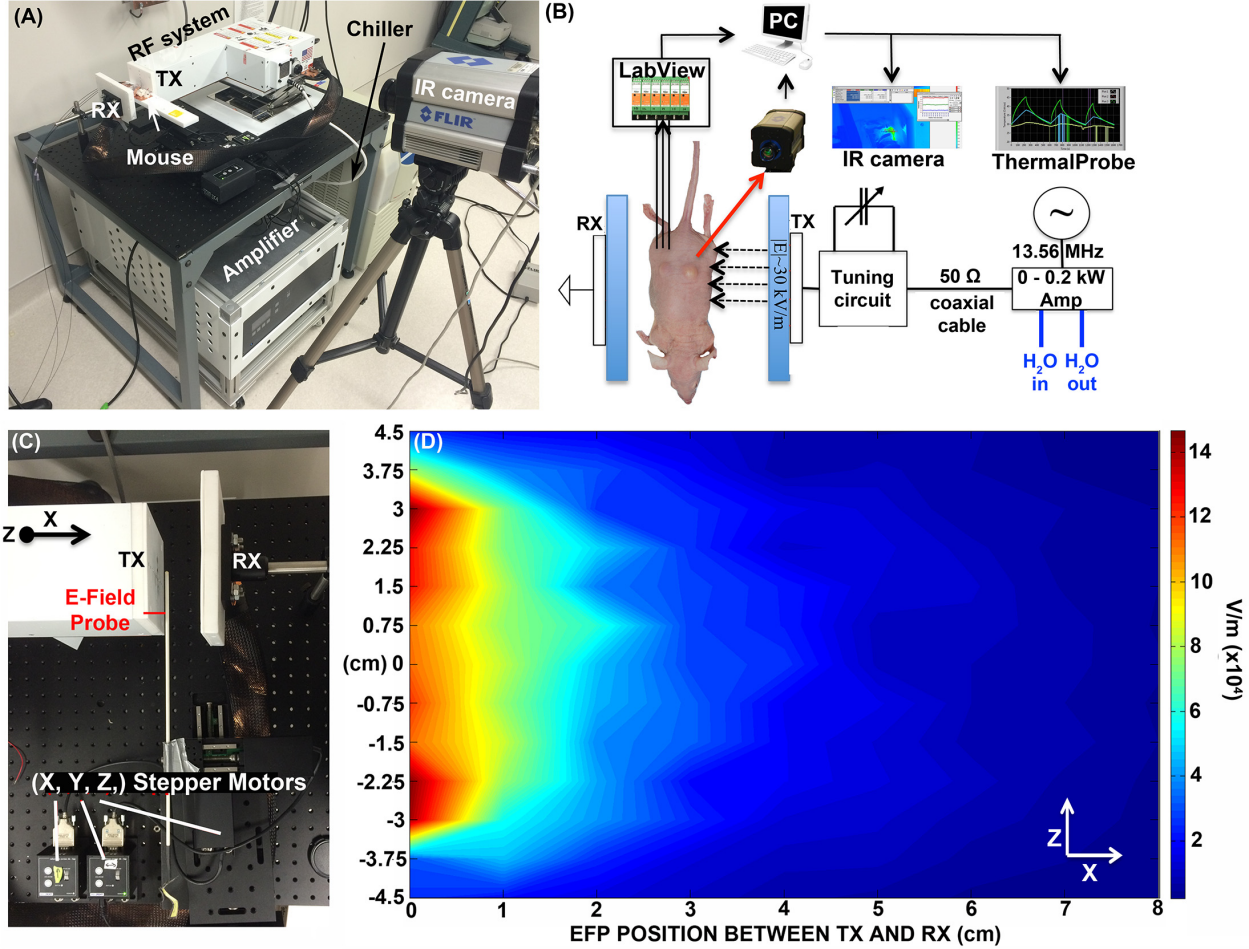
Portable RF system setup and generated electric field. (A) Portable RF system consists of the transmitting unit (TX) and receiving head (RX) that generates a high-power electric field across the specimen (e.g. mouse). The system is driven by a variable power fixed RF amplifier (0–200 W, 13.56 MHz) that is cooled during operation by a water chiller. Heat production is monitored using an infrared (IR) camera or direct insertion of fiber optical probes. (B) Circuit representation of the portable RF system. (C) Setup for extracting electric-field intensities. An electric-field probe (EFP) is placed at specific points along the x- and z-axis in between the TX and RX heads and measures the voltage at each point for 20 W RF-power. (D) The electric field is derived from the voltage data and is plotted as an intensity contour plot.

A photograph of the portable-RF system (p-RF) system alongside a schematic representation of the p-RF experimental setup is illustrated in **Fig 1A and 1B**. Full dimensions can be found in **S1 Fig** The device is powered by a 200 W fixed-frequency (13.56 MHz) water-cooled power supply (Seren, RX01/LX01 Series, Industrial Power Systems, Inc.), which is connected via a high-current carrying capacity 50 Ω co-axial cable. The specimen to be exposed to RF is placed between the transmitting and receiving heads (TX and RX, respectively). Increases in temperature are recorded using either a 1 mm outer diameter fiber optic Teflon coated thermal probes (Photon Control, Canada), with a temperature accuracy of ± 0.5°C, or an infrared (IR) camera (FLIR SC 6000, FLIR Systems, Inc., Boston, MA), with a temperature accuracy of ± 2°C (640 × 512 resolution InSb detector with a mid-wavelength IR spectral range of 3.0–5.0 μm). Thermal probe data is captured using a custom built LabVIEW Virtual Instrument (National Instruments, Austin, TX). The generated RF electric-field was characterized using a Teflon coated electric field probe (TherMed, LLC, Erie, PA) attached to an adjustable x,y,z stage (Thorlabs, Inc.) for adjustable positioning, as shown in **Fig 1C and 1D**. Full details for electric-field measurements can be found in **S2 Fig** As can be seen in **Fig 1D**, the ‘active’ area of RF electric-field exposure is centered ~6 cm around the mid-point of the TX head and extends ~1–2 cm across the x-axis, causing a heating profile that is gradually reduced as the sample is located further away from the TX head.

The p-RF system itself is relatively small (length ~ 60 cm) when compared to our larger RF models [1, 7, 14]. Similar to our previous RF generators, the design delivers a strong alternating (13.56 MHz) electric field across the TX and RX heads [29] using a cascade LC network. However, unlike our previous systems, this system is not capacitively-coupled and does not model an ideal parallel-plate capacitor configuration where the electric field would be approximately uniform across the TX and RX heads. Instead, this system transmits an electric-field which gradually diminishes across the TX-RX heads and is hence classified as an “end-fired transmission configuration”.

### IVM-RF system

**Fig 2:**
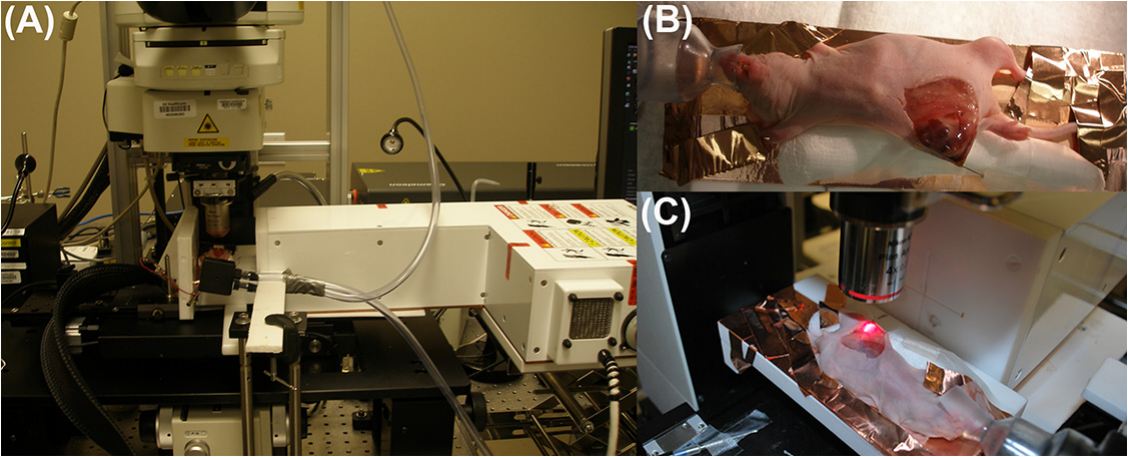
Portable RF system retrofitted to the IVM. (A) The RF system integrated into the intravital microscope (IVM) for real-time imaging under RF exposure. (B) Mouse manipulation for imaging–an incision is made to expose and gently manipulate the 4T1 tumor for IVM imaging. (C) 4T1 tumor under IVM illumination with a x4 objective lens.

A picture of the p-RF system retrofitted to a Nikon A1R^+^ IVM is shown in **Fig 2A**. The Nikon A1R^+^ is a laser scanning confocal microscope equipped with two scanning mechanisms, a conventional galvanometer driven system and a resonant scanner. The A1R^+^ is equipped with 4 solid-state lasers (405, 488, 561 and 640 nm) and 4 fluorescence detectors, including two GaAsP PMTs. The A1R^+^ is also equipped with a large platform motorized stage (Prior Scientific ZDeck) and a collection of long-working distance objectives ranging from low magnification, large field of view (4x 0.2NA and 10x 0.4 NA), up to high resolution, water immersion (16x 0.8 NA and 25x 1.2NA) lenses. System operation and image acquisition are controlled by Nikon NIS Elements software (v 4.0). Once the RF instrument was fitted to the IVM, our initial evaluation of the integrated system involved gradually increasing the p-RF power (without a sample) whilst monitoring the voltage induced across the IVM chassis by connecting an oscilloscope probe to the electrode ground pins located behind the objective lens on the IVM system. At all power levels, including the highest power of 200 W RF, the voltage induced on the chassis was less than 500 mV, which is deemed negligible and was not predicted to interfere with hardware. This test procedure was performed to make sure the RF energy was not directly coupling to the IVM microscope, which would most likely cause irreversible electronic and structural damage to the IVM system. Minor interferences included software malfunction in the form of randomly opened browser windows and text appearances–we termed this effect ‘ghost writer’ and discovered the origin of this effect to be due to RF fields coupling to the computer keyboard. Wrapping the keyboard cable around a ferrite core balun to reduce RF interference solved this problem. We also observed interference with the motorized stage, which was solved by insulating the joystick controller box with aluminum foil.

### Animal models

Nude mice (4–6 wk old) were obtained from Charles River Laboratories, Inc. (Wilmington, MA). Breast tumors were established using fluorescent 4T1 td-Tomato Bioware Ultra Red mouse mammary cancer cells purchased from Caliper Life Sciences (Hopkinton, MA). Mice were treated and imaged when tumors reached a size ~ 8 to 10 mm in diameter. At termination of the imaging session, animals were euthanized via CO_2_ exposure followed by cervical dislocation. All procedures were performed in accordance with protocols approved by the Institutional Animal Care and Use Committee at Houston Methodist Research Institute and according to the NIH Guide for the Care and Use of Laboratory Animals.

### RF-IVM animal manipulations

Mice bearing 4T1 tumors were exposed by a small midline incision whereby the fascia between the skin and muscle was disrupted using a cotton swab. An inverted skin flap was elevated using rolled cotton gauze. Images of the mice being manipulated for RF-IVM are shown in **Fig 2B and 2C**. Mice were anesthetized using 2–3% isoflurane (Aerrane; Baxter Healthcare, Deerfield, IL, USA) administered through an isoflurane vaporizer system (E-Z Systems, Palmer, PA, USA). Mice were kept on a warming pad during surgical preparation and imaging experiments to maintain core body temperature. During the image recordings the tumor was continuously moistened with saline and the temperature was monitored using Teflon coated fiber optic probes and/or an IR camera. For imaging with water immersion lenses, a coverslip was gently positioned on top of the moistened imaging area using a manual micromanipulator (Kite, WPI). Time-lapse recordings were captured on selected fields of view at frame rates of 10–30 fps.

### Fluorescent tracers

The fluorescent tracers used in this experiments were Albumin-Alexa Fluor 647 (MW~ 66kDa) and fluorescein isothiocyanate-dextran (FITC-dextran, MW~70 kDa). Both were obtained from Life Technologies, Grand Island, NY. Mice were given 50 μl retro-orbital injections of either Alexa 647 or FITC-Dextran (or both) at concentrations of 10 mg/Kg (suspended in phosphate buffered saline, PBS). The mice were then subjected to RF exposure with or without simultaneous IVM imaging. Fluorescent tracers were used in this study to contrast the tumor blood vessels and to investigate extravasation, due to increased vascular permeability, and the diffusion of the tracers into the tumors. Cancer cells were identified by their expression of tdTomato-fluorescent protein. FITC-dextran, Td-Tomato and Albumin-647 fluorescence signals were detected sequentially using laser excitation lines at 488, 561 and 640 nm, while emission was recorded using narrow band pass filters (30–50 nm bandwidth) at 520, 600 and longer than 640 nm, respectively. Three channel images were captured a 512x512 frame sizes with pinhole diameters set at 1 Airy unit (AU) calculated at 561 nm.

### Immunofluorescent imaging

The complete macro-perfusion and uptake of fluorescent tracers throughout the tumor in RF and non-RF treated mice was analyzed *ex vivo* using immunofluorescence imaging. Tumor blood vessels were visualized using antibodies for CD31 to assess tissue penetration by extravasated albumin or FITC-dextran. Frozen tumor sections were fixed with 4% paraformaldehyde, blocked with 5% normal horse serum and 1% normal goat serum in PBS, and immunofluorescently stained using antibodies to CD31 (BD Biosciences, San Jose, CA). Sections were then incubated with goat anti-rat IgG Alexa Fluor 488 antibody (Jackson ImmunoResearch, West Groove, PA)[30]. The images were captured using our Nikon A1R^+^ confocal microscope and analyzed using Nikon NIS-Elements AR software (v3.2). The ratio of pixels in the whole image that has higher fluorescence intensity than the threshold (background) was shown as positive area fraction[31, 32]. The data were shown as the average ± SD from representative sections of more than 5 images of tumors.

### Algorithms for quantifying fluorescent tracer perfusion

To quantitate the fluorescent tracer accumulation in the tumor and the extravasation from blood vessels, we used a simple algorithm based on global threshold segmentation and binary masking techniques applied to the images acquired in live animals. By thresholding the Td-Tomato fluorescence component, we first create a binary image, which is used to generate a mask for the tumor. Dilate and erode operations are used to remove holes and smooth the edges of this mask. A similar method is used to create a vasculature mask based on the high intensity values of the FITC-Dextran or albumin-647 signals. The two masks are then combined to find the extravascular component of the tumor area and this resulting mask is used to quantify the amount of tracer dye, which has migrated into the tumor.

## Results and Discussion

### Tumor temperature modulation

**Fig 3:**
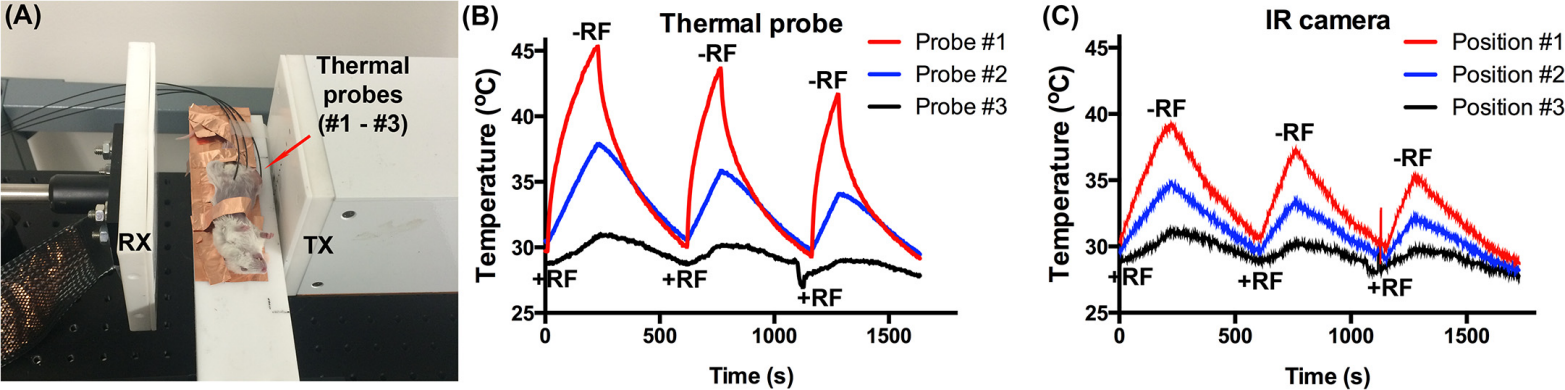
Modulation of tumor temperature using RF exposure. (A) Thermal fiber optic probe placement. Probes #1–3 are positioned (i) under the skin but above the tumor; (ii) under the skin in between the tumor and the main body; and (iii) under the skin next to the intraperitoneal cavity. (B) Extracted thermal probe data. The recorded temperature of the probes was modulated by turning on and off the RF system (+RF and–RF). The system was turned off once the tumor temperature (probe #1) reached 45°C, 43°C, and 41°C, respectively, and was turned on when all probes had values in the range ~29–31°C. (C) The IR camera simultaneously measured the surface temperature of the points where the thermal probes were located.

Initial testing of the RF-IVM system included exposure of a 4T1 tumor-bearing mouse to RF energy, without imaging, to confirm tumor heating. **Fig 3A** depicts the experimental setup. The mouse was placed on a specially designed Teflon stage covered with a thin film of copper tape to electrically ground the animal: preventing surface electrical charge accumulation that could cause thermal injury. The animal loaded stage was placed between the TX and RX heads of the p-RF system. Three fiber optic thermal probes were directly inserted into the mouse at different positions surrounding the tumor and at unique distances from the TX head. Probe #1 (closest to the TX head) was inserted under the skin but above the tumor mass; probe #2 was inserted under the skin in between the area where the tumor is projected from the main body of the mouse; and probe #3 was inserted above the exposed intraperitoneal cavity. Given that tissue located near probe #1 would likely heat the greatest due to its proximity to the TX head, we used this as a reference in turning on and off the RF system at different temperature points: 45°C, 43°C and 41°C. The tissue temperature was cooled down to around ~30°C (due to the air conditioned operating room) between each RF exposure. The total power needed to generate these heating profiles was 90 W. As seen in **Fig 3B,** the tumor temperature initially increased from 30°C to 45°C in ~250 s, taking ~375 s to cool back down to 30°C. At this point the RF was turned back on and the tumor was heated to 43°C before being turned off again. This was repeated to a final tumor temperature of 41°C. The temperature data from probes #1–#3 demonstrated a reduction in tissue heating due to the fall off in electric-field intensity from the TX head. If the electric field were to be constant across the TX and RX heads, such as that approaching the condition of an ideal parallel plate capacitor model, then any fluctuations and variations in temperature would most likely be attributed to differences in permittivity and conductivity between the tissues, organs, and tumors of the mouse, as will be discussed.

The electric field intensity around the tumor as well as the tumors’ dielectric properties are perhaps the two most important physical parameters governing the heating rates of individual tumors. Dielectric in this case refers to how much electrical energy a material will absorb and convert to heat, and is frequency dependent. A recent publication demonstrated anti-tumor effects resulting from non-invasive RF[1]. In their study, Raoof *et al*. subjected mice bearing orthotopic-implanted human hepatocellular and pancreatic xenografts to weekly RF exposures. Their results indicated that RF-alone was enough to cause an anti-tumor effect in hepatocellular carcinomas and could be explained purely on the principle of the tumors’ dielectric properties being larger than normal, healthy tissues. The ability of a material to store and dissipate electrical energy as heat can be described by the real (*ε’*) and imaginary (*ε”*) parts of the complex permittivity function (ε*). This relationship is given by Eq 1:

$$\varepsilon^{*}\left(\omega \right)=\;\varepsilon \prime \left(\omega \right)-\;i\varepsilon \prime \prime \left(\omega \right)$$ (1)

where *ω* is the radial frequency (*2πf*). The real term of Eq 1 gives information as to how much electrical energy can be stored in a material whilst the imaginary term denotes how much of this energy is converted to heat.

In a purely ideal clinical situation, the imaginary values for tumor tissues would be significantly higher than that of normal, healthy tissues, whereby the tumor would heat rapidly up to temperatures that induce either hyperthermia (leading to natural programmed cell death mechanisms) or complete ablation and necrosis. The dielectric properties of both cancerous and normal tissues were measured by Raoof *et al*. (using a permittivity analyzer), and were shown to be larger for tumors than normal cells. The relationship between a material’s permittivity and its effect on heat production when exposed to a time varying electric-field is given by the following equation:

$$HR=\;\frac{d\text{T}}{dt}\;=\frac{\varepsilon_{0}\varepsilon^{"}\left(\omega \right){\left|E\right|}^{2}}{2\rho c_{p}}$$ (2)

where *ε*_*0*_ is the vacuum permittivity, *ε”* is the imaginary part of the complex permittivity, *E* is the electric field intensity in the sample, *ρ* is the density, and *c*_*p*_ the specific heat capacity. In this fundamental governing equation, all of the relevant physical variables are contained that describe how a sample will respond to exposure to an electric field. This equation, especially the strong dependency on electric field intensity, can help further explain the decrease in heat production: the temperature probes are located further away from the TX head with the electric-field strength gradually decreasing.

**Fig 4:**
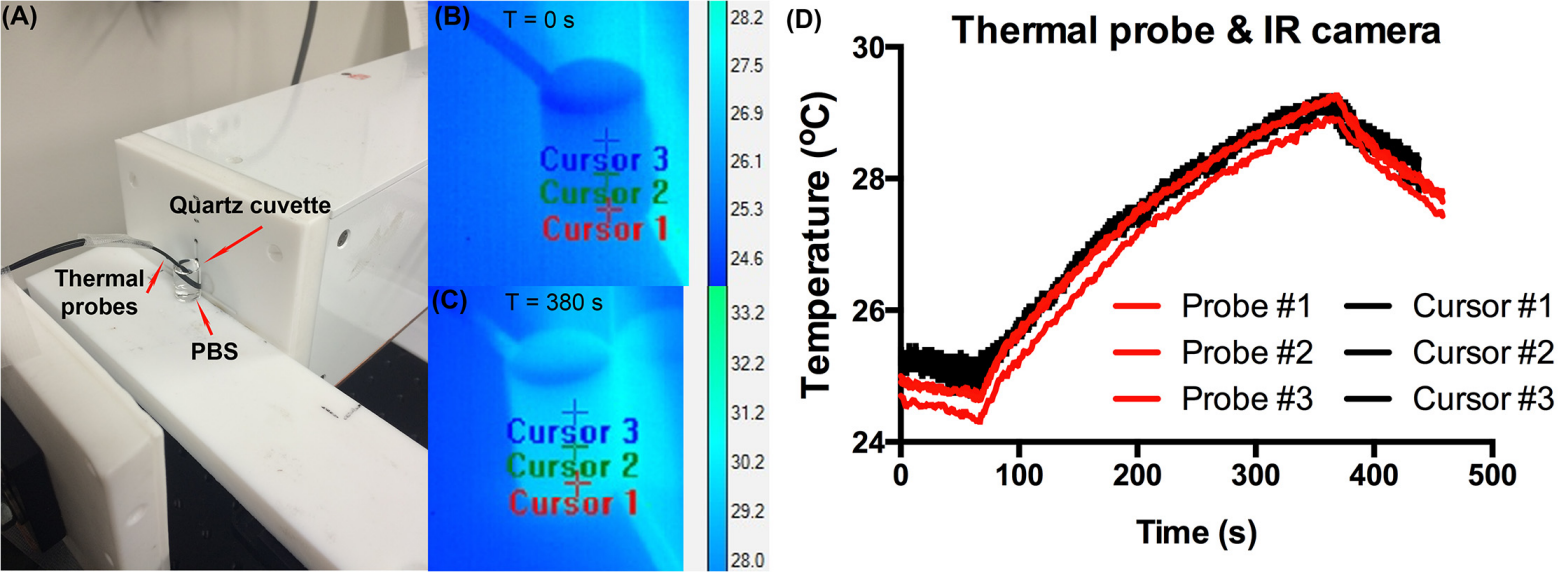
Thermal probe and IR camera calibration. (A) Three thermal probes were places in a quartz cuvette filled with phosphate buffered saline (PBS) and exposed to 200 W of RF. The IR camera captured the surface temperature of cursor points located next to the thermal probes for the RF exposure time 0 s—380 s (B and C, respectively). (D) Comparison of thermal probe and IR camera heating data.

In this study, the surface temperature of the locations in which the thermal probes are located were also captured using an IR camera, as shown in **Fig 3C**. As can be seen, there are significant similarities and differences when compared to the thermal probe data. The IR data indicates a decrease in final tissue temperature compared to the probe #1 measurement of ~5°C, and a decrease in temperature of ~3°C for probe #2. The temperatures are similar for probe #3. To further test the differences and error margin between the IR camera and thermal probe data, all three probes were immersed in 1.3 ml of PBS contained in a quartz cuvette and exposed to the RF field. The temperature data is shown in **Fig 4**. There is a close match between the recorded IR camera and thermal probe data with an error margin between 0.2–0.5°C. This similarity was expected as the quartz cuvette is almost optically transparent across the IR wavelength range 3.0–5.0 μm. Given the close similarities between the IR camera and thermal probe data, the differences in mice heating shown in **Fig 3** is most likely due to mismatch between the probe placement and IR cursor location. For example, the position of probe #1 is actually deeper under the skin of the mouse than probe #3 (as well as being closer to the tumor) so will likely show larger heat production due to the heating of the tumor when compared to the surface IR camera measurements. Also, surface measurements are generally likely to be lower than inter-tissue temperatures due to the cooling effect from the room temperature environment. Finally, optical losses and absorption of propagating IR energy through the skin will most likely reduce the intensity of the IR photons on the surface of the mouse, which are being detected using the IR camera.

### Multi-channel IVM-RF imaging and high-temperature vessel degradation

**Fig 5:**
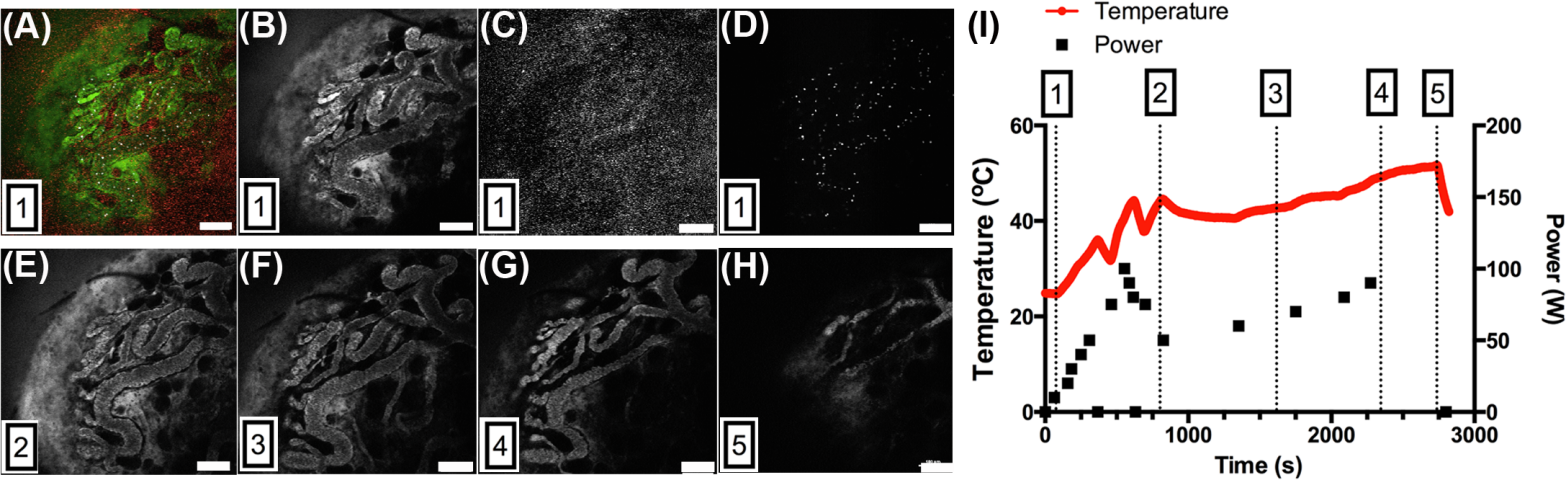
Multi-Channel IVM-RF imaging. (A) Overlay of the independent IVM channels (FITC, Texas Red, and Cy5). (B) Tumor vessels are highlighted using FITC-dextran fluorescent tracers, (C) Fluorescent emission from the transfected 4T1 tumor cell line, (D) Cy5 emission from the DiD-stained Red Blood Cells. Figure (A)–(D) were taken at time = 78 s. Figure (E)–(H) depict the FITC channel (vessels) at different time points: 762, 1650, 2382, and 2742 s, respectively. Figure I illustrate the tumor temperature with respect to time and applied RF power. The numbers 1–5 shown in the bottom left hand side of each figure correspond to the 5 different time-points highlighted in Figure I.

**Fig 5** depicts real-time multi-channel IVM-RF imaging on an exposed 4T1 tumor. Three separate channels were imaged: FITC (vessels with dextran), Texas Red (4T1 transfected tumor), and Cy5 (red blood cells, RBCs). **Fig 5A** shows the merged channels, whilst the individual channels are shown in **Fig 5B–5D**. **Fig 5E–5H** shows changes in the vessel architecture for four different time points, illustrated as time point 1 through 5 in **Fig 5I** (NB: time point number 1 corresponds to imaging before the addition of RF exposure). Also shown in **Fig 5I** is the graph of tumor temperature and RF power versus time. Tumor temperature in this case was monitored using a temperature probe placed in the tumor. A time-compressed movie of these merged and individual channels can be found in the **S1 Movie.**

As can be seen from these results, the tumor vessels start to narrow and constrict once the tumor temperature is raised above 41°C. At a final tumor temperature of 44°C, the intravascular cells are completely stagnant and the vessels have stopped functioning. This can also be seen in the **S1 Movie** with regards to the flow of red blood cells. Once the temperature is elevated above 41°C the flow of RBCs becomes irregular and there are some vessel compartments where blood flow has ceased completely. Note, with regards to the time points where the RF power was intermittently terminated in order to prevent overheating of the tumor. The use of a ramped heating profile enables us to tailor off the power once the designated temperature is reached so that an accurate set temperature can be maintained. This can be seen from time points 2 and 3 where the power is rapidly decreased then gradually increased to allow for a more gentle heating profile.

**Fig 6:**
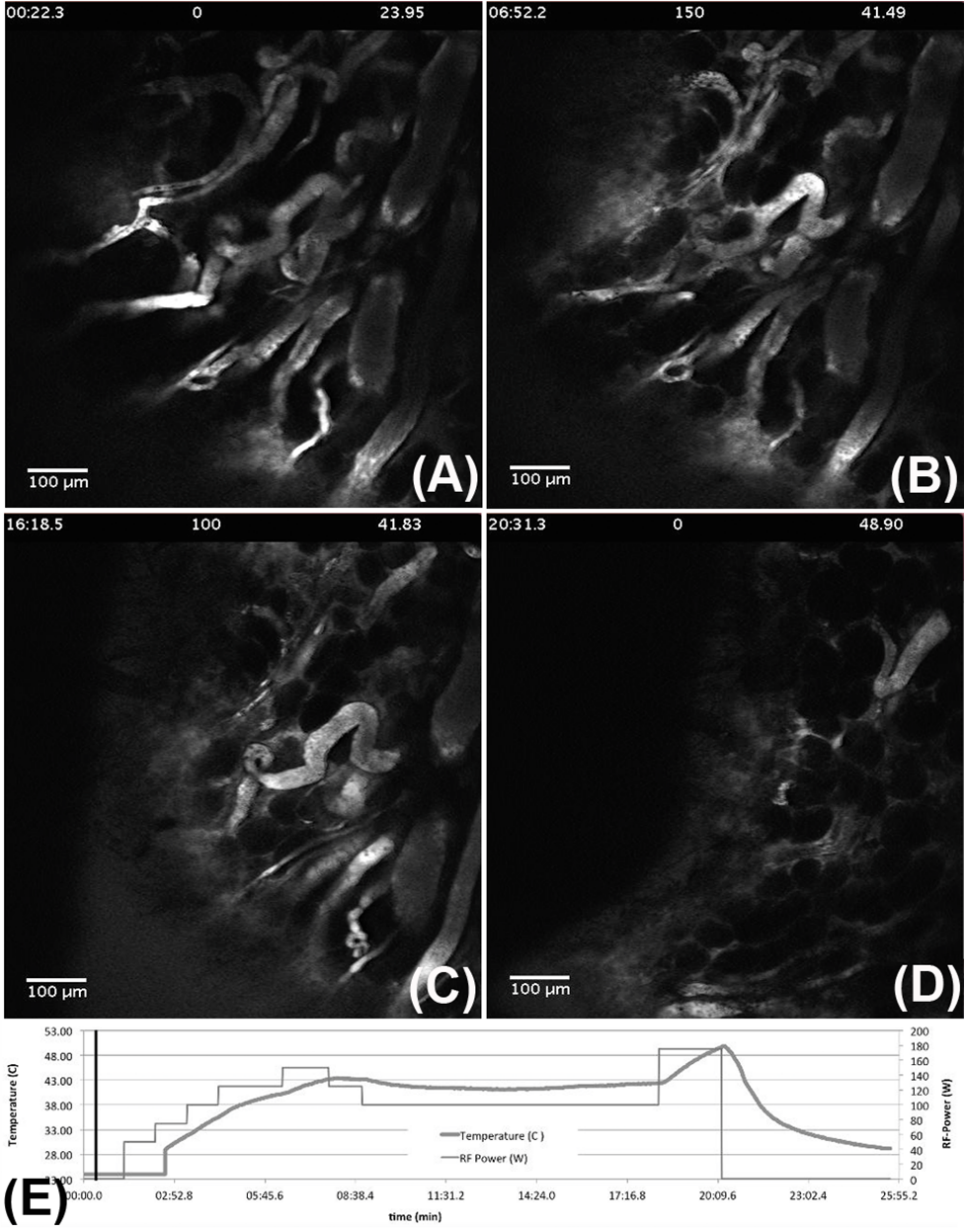
High-temperature vessel degradation. (A)–(D) Impact of RF exposure on vessel architecture at four different time-points: 0:22, 6:53, 16:18, and 20:31 minutes, respectively. The tumor temperatures and RF power at those time points are shown in the upper-middle and upper-right hand side sections, respectively. Figure (E) illustrates the change in temperature and power with respect to time. Vessel degradation can be seen for temperatures > 41°C. A complete breakdown of the vessel architecture can be seen for temperatures > 47°C.

A repeat of this experiment (but without the RBC staining) is shown in **Fig 6**. The effect of vessel degradation is more pronounced in these images. By looking at the four different time points it can be seen that some low level of vessel degradation is evident for temperatures between 41.5–41.8°C (we held this temperature range for ~10 minutes). After this, upon application of more RF power, the increase in tumor heat production (up to ~49°C) resulted in severe degradation and complete shutdown of the tumor vessels. A full movie of these effects can be seen in **S2 Movie**. The results shown in Figs 5 and 6 illustrate the effect of high temperatures on vessel architecture and RBC flow dynamics. Although it is well known that vessel damage can occur for temperatures greater than 41°C, it has been demonstrated that enhanced vessel permeability and perfusion of circulating macromolecules, chemotherapeutics, and drugs can be expected for temperatures across the range 39°C–41°C (the referenced review article by Roussakow offers a comprehensive and concise overview of the field[33]).

### RF-induced fluorescent tracer transport and perfusion into 4T1 tumors

**Fig 7:**
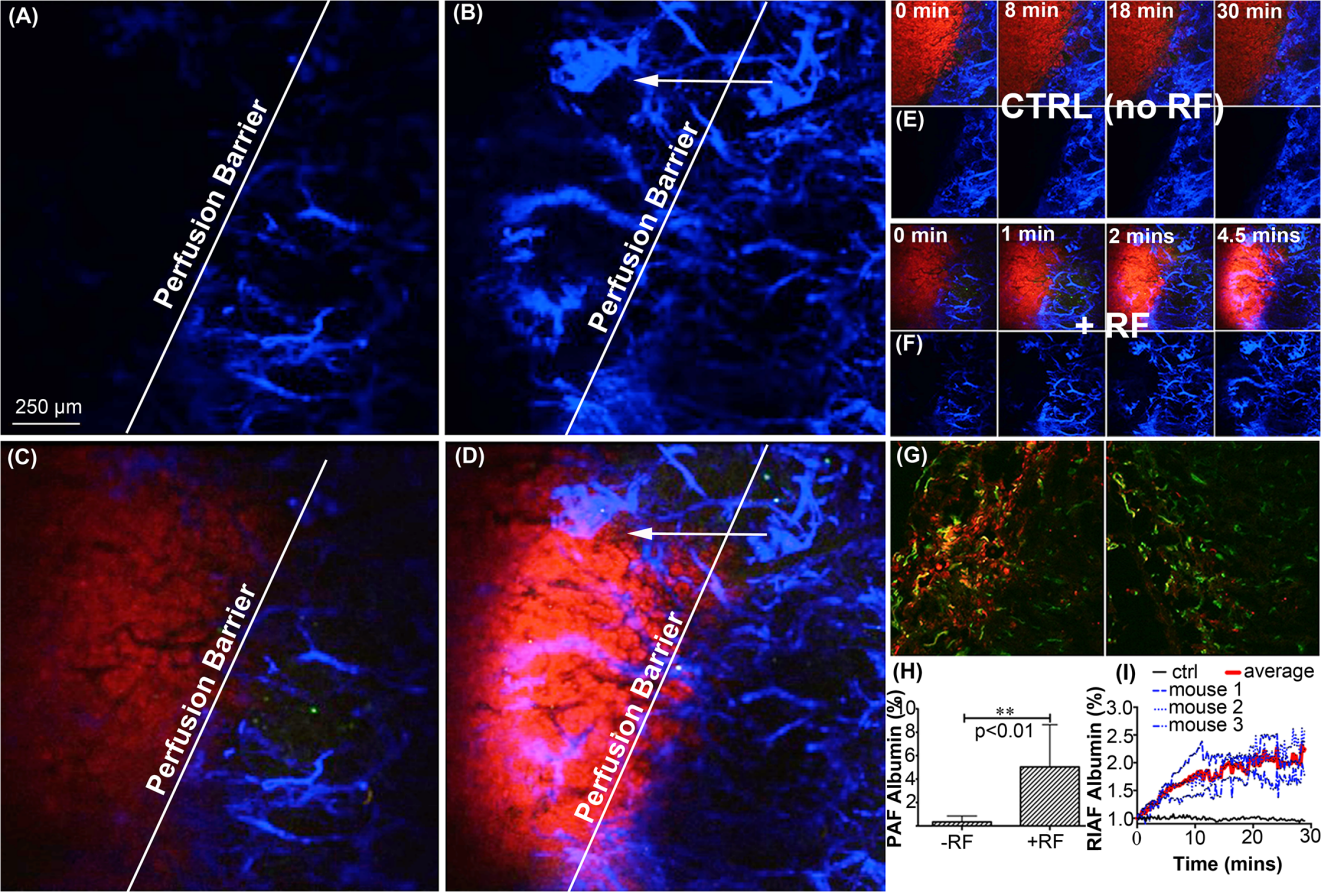
Real-time RF-IVM imaging and post capture analysis. RF exposure shows transport of fluorescently bound albumin across the perfusion barrier into tumor region. Figure (A) and (B) depict the blue image channel (albumin) before and after (4.5 min) RF exposure. This data is shown superimposed with the tumor (red) channel in Figure (C) and (D). Figure (E) Control mouse (no RF) was imaged for 30 minutes on both channels. There is no transport of albumin into the tumor across the perfusion barrier. (F) Time lapsed images of the data shown in Figure (A) and (B). Figure (G) 4T1 tumor slices immunohistologically stained to the antibodies CD31 (green, vasculature endothelial cells), and albumin (red) for both RF (left image) and non-RF (right image) groups. Figure (H) depicts positive area fraction (PAF) of albumin accumulation in tumor slices. Finally, (I) is a quantitative video analysis of relative increase in albumin fluorescence (RAIF) in multiple 4T1 tumor surfaces exposed to RF under IVM (n = 4).

Mice with 4T1 tumors were surgically prepared for RF-IVM as described in the methods section. Mice received intravenous injections of 50 μl albumin-alexa-fluor 647 dye (10 mg/Kg) via retro-orbital injection and imaged with and without RF (as a control). For all experiments the RF was turned off once the tumor temperature reached 41°C (unless otherwise stated) as indicated by the IR camera. **Fig 7A–7D** depict perfusion of the albumin tracer out of blood vessels and into the tumor during an RF treatment duration of 4.5 minutes. Enhanced tumor perfusion is particularly evident when comparing **Fig 7A and 7B** for the start and end-points (0 and 4.5 minutes, respectively) for the albumin only (blue) channel. The complete video files (edited to remove image jitter due to mouse respiration) are in **S3 and S4 Movies**. For a control, the same experiment was imaged without RF exposure (**Fig 7E**) for 30 minutes. An impaired perfusion barrier is evident as no albumin penetrating into the tumor during the imaging session (30 min, **Fig 7E**). This impaired perfusion is characteristic of tumors due to high pressure, chaotic vasculature, and the resulting tumor vessel compression [34, 35]. Limited vascular perfusion was observed in multiple mice during imaging sessions lasting up to one hour.

Immunofluorescent staining was performed on RF and non-RF treated tumors (n = 6), as described in the methods section, to visualize the distribution of the albumin tracer from the vessels (**Fig 7G)**. Tissue accumulation of albumin (seen as red) occurs in close proximity to vasculature endothelial cells (seen as green) and is most evident for the tumors treated with RF (left-hand image in **Fig 7G**). The ratio of red pixels greater than the threshold (background) over total pixels was shown as a positive area fraction (PAF) in the graph in **Fig 7H**. The data indicates a five-fold enhancement of albumin perfusion into the tumor for RF-treated mice compared to controls. Using an image analysis algorithm (methods section) to quantitate the surface perfusion of fluorescent tracers into tumor tissue directly from the real-time IVM imaging data (**Fig 7I**) we found on average a doubling of surface perfusion of the fluorescent tracers in the tumors when compared to the controls.

### Prolonged RF-induced biological effects

**Fig 8:**
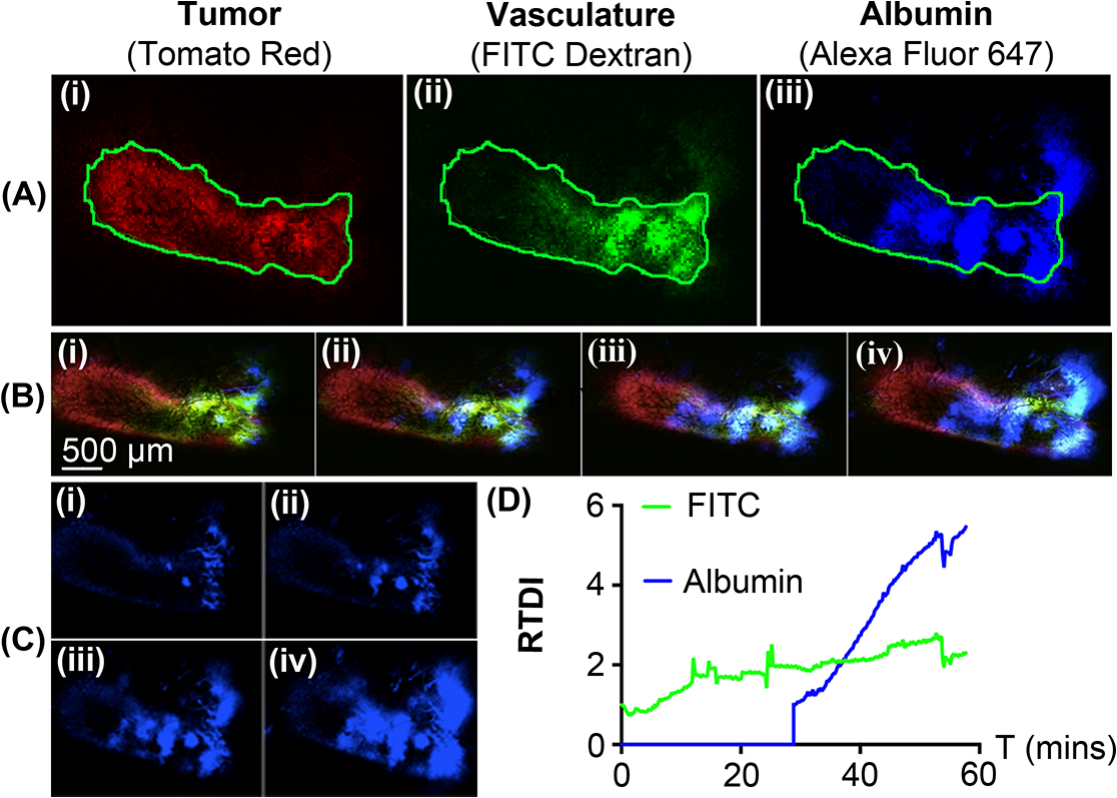
Post-RF IVM analysis of images using template and masking algorithms. FITC-dextran was injected followed by 30 mins RF exposure. Albumin was then injected and imaged for 30 minutes without RF exposure. (**A**) The tumor area (i) is demarcated using a green line and allows FITC-dextran (ii) and albumin (iii) perfusion to be monitored. (**B**) These masks are applied to the full time-lapsed video for all channels. (**C**) Areas where both albumin and FITC-dextran overlap are processed to quantify the relative average intensity of albumin perfusion after 30 mins of RF exposure. (**D**) Relative tumor dye intensity (RTDI) versus time. The intensity of FITC-dextran gradually increases over the 30 min of RF exposure and continues for another 30 mins after the RF is turned off. The intensity of albumin increases once injected after the RF is turned off (t = 30 mins) and continues for 30 minutes. This suggests RF-mediated effects are prevalent even after RF exposure.

In order to see if the tumor perfusion of fluorescent tracers is evident after RF exposure we performed further experiments whereby fluorescent tracers were administered after RF exposure. Mice bearing 4T1 tumors were administered 50 μl FITC-dextran (10 mg/Kg) via retro-orbital injection, and exposed to RF for 30 minutes. After RF exposure, we then administered 50 μl albumin (10 mg/Kg) and performed IVM imaging for another 25 minutes, without RF exposure. These results (as well as the technique used for analysis) are shown in **Fig 8**. The movie file of this time-lapse experiment can also be found in the **S5 Movie**. The relative tumor dye intensity (RTDI) for the FITC signal (as shown in **Fig 8D**) is shown to gradually increase over time and this continuous rate of increase persists even after RF exposure. This is also true for albumin, which shows a rapid increase in RTDI once administered immediately after RF exposure. This suggests that the effects of RF energy on the tumor vasculature system enabling increased perfusion of fluorescent tracers into the tumor are long-lived, at least for up to 25 minutes after RF exposure. Given that both tracers are of similar molecular weight (albumin = 66 kDa, FITC-dextran = 70 kDa) it is interesting to see an increase in perfusion of albumin over the FITC-dextran. This could be due to a variety of reasons such as: heating effects on the structure of dextrans, the solubility of dextrans versus albumin, the chemical and biological interaction of dexrans and albumins in the tumor microenvironment, etc. More work is currently being undertaken to understand these size-related effects.

## Conclusion

We have designed and engineered a custom portable RF system, which can easily be retrofitted to a high-resolution Nikon A1R^+^ intravital microscopy system enabling *real-time in vivo* image acquisition of RF-induced biological effects such as enhanced fluorescent tracer perfusion into tumors and high-temperature vessel deformation. The integration of a high-power RF generator did not result in hardware or electronic damage as may be feared in the presence of high magnitude electric fields. The use of fiber optic thermal probes and an infrared imaging camera enabled us to verify that tumor temperatures were successfully modulated by the RF system. Short durations of RF exposure resulting in temperature elevations below 41°C were shown to increase the perfusion and delivery of Alexa-647-albumin and FITC-dextran fluorescent tracers into the tumors. For tumor temperatures above 41°C, slight vessel degradation was evident in exposed tumors, with complete vessel deformation and shutdown occurring for temperatures greater than 44°C. Future work with the new imaging platform include detailed analysis of RF-mediated biological effects, including vascular permeability, blood flow, tissue alterations, nanoparticle and drug accumulation, tissue penetration, and cellular migration events. These results highlight the use of our integrated IVM-RF imaging platform as a powerful new tool to visualize the dynamics and interplay between radiofrequency energy and biological tissues.

## Supporting Information

S1 Fig Portable RF system dimensions (inches). (DOCX)

S2 Fig Electric Field Probe Measurements. (A) A custom-made electric field probe (EFP, Thermed Inc, PA, USA) was used to measure voltages in between the transmitting (TX) and receiving (RX) heads of the portable RF-probe (p-RF) via an oscilloscope. The EFP was attached to three stepper motors that could manipulate the probe across the x, y, z-axis. The plane shown in the upper left section of (B) was fully characterized across a TX:RX distance of 8 cm across the full z-axis -4.5 cm to +4.5 cm (NB: the voltages measured lie on the plane perpendicular to the figure). The extracted voltages were then fitted as a contour plot in Matlab and are shown in (C). The direction of the voltage plane is also shown in the bottom right-hand corner. As can be seen, the voltage is concentric around the 0-point and gradually falls off as the distance between the probe and the TX head increases. The voltages were characterized for 20 W, rather than 200 W, as the probe would heat significantly if allowed to go above 40 W and would damage the probe. Also, 8 cm was chosen as this was the TX:RX distance used in all experiments Converting Voltages to Electric fields. The component of electric field in any direction is the negative of rate of change of the potential in that direction. If the differential voltage change is calculated along a direction dx, then it is seen to be equal to the electric field component in that direction times the distance dx. The electric field can then be expressed as E = -dV/dx. Using the voltages extracted above and the distances between each measured voltage (0.01 m) allowed us to calculate the electric-field contour plot shown in the main text (Fig 2D).
(DOCX)

S1 Movie In vivo multi-channel IVM-RF imaging. The channels from left to right are (i) all channels merged, (ii) FITC (tumor vessels), (iii) Texas Red (4T1 tumor), and (iv) Cy5 (DiD Red Blood Cell staining). The upper left hand side of each frame illustrates the time. The temperature and power versus time graph is shown in Fig 5.
(MP4)

S2 Movie High-temperature vessel degradation. In vivo imaging of vessel degradation due to RF-induced elevated temperatures. The left frame is a 2 minute loop of the tumor vessel (FITC) before RF imaging (control) and the right frame depicts changes in tumor vessel architecture due to high temperature. The bottom frame illustrates tumor temperature and power with respect to time (created using ImageJ).
(MP4)

S3 Movie Enhanced Perfusion of Albumin into breast Tumor- All IVM Channels. Mice were administered albumin (blue) via retro-orbital injection and exposed to RF for 5 minutes. Enhanced perfusion of albumin into 4T1 breast tumor (red) is evident within the first few minutes of exposure.
(MOV)

S4 Movie Enhanced Perfusion of Albumin into breast Tumor: Blue IVM Channel. Mice were administered albumin (blue) via retro-orbital injection and exposed to RF for 5 minutes. Enhanced perfusion of albumin into 4T1 breast tumor (dark black circle area) is evident within the first few minutes of exposure. Refer to Video 1 for tumor (red) channel.
(MOV)

S5 Movie Albumin perfusion out of tumor vasculature system. Time-Lapsed movie of Albumin (blue) perfusion into tumor (red) after the mouse was subjected to 30 mins of RF exposure. Time period was 30 minutes. The mouse was previously exposed to RF for 30 mins with FITC-Dextran (green) to stain the vasculature system. This suggests that the effects of RF are still evident after RF exposure.
(MOV)
