# Supplementary figures and images for: A New Imaging Platform for Visualizing Biological Effects of Non-Invasive Radiofrequency Electric-Field Cancer Hyperthermia
Source: PLoS One. 2015 Aug 26;10(8):e0136382. doi: 10.1371/journal.pone.0136382 (PMC4550384; doi:10.1371/journal.pone.0136382)

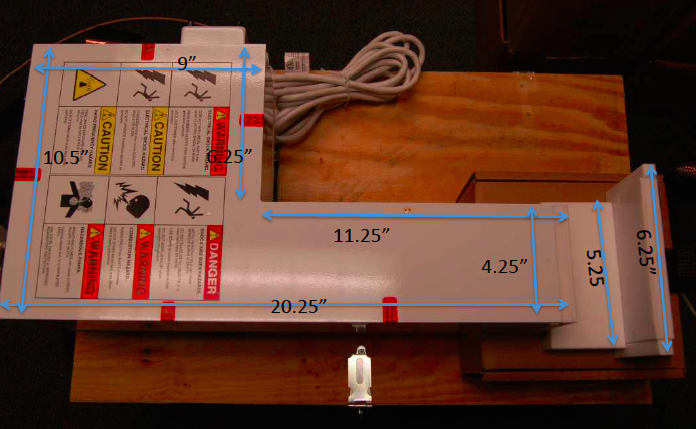


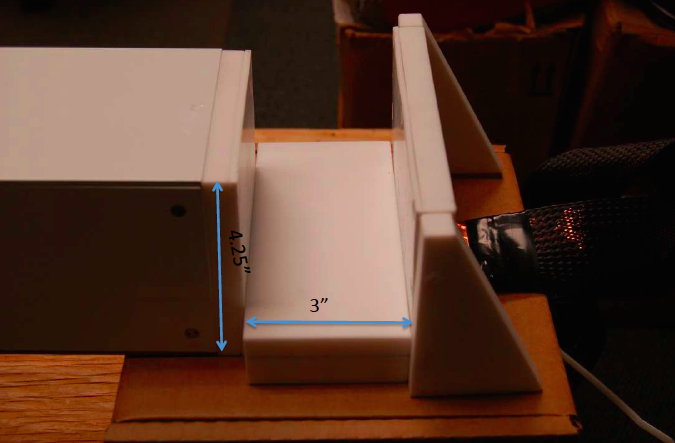


**S1. Fig.** **Portable RF system stage dimensions** (inches).

Supplement: S1 Fig — (DOCX) [file pone.0136382.s001.docx]

**
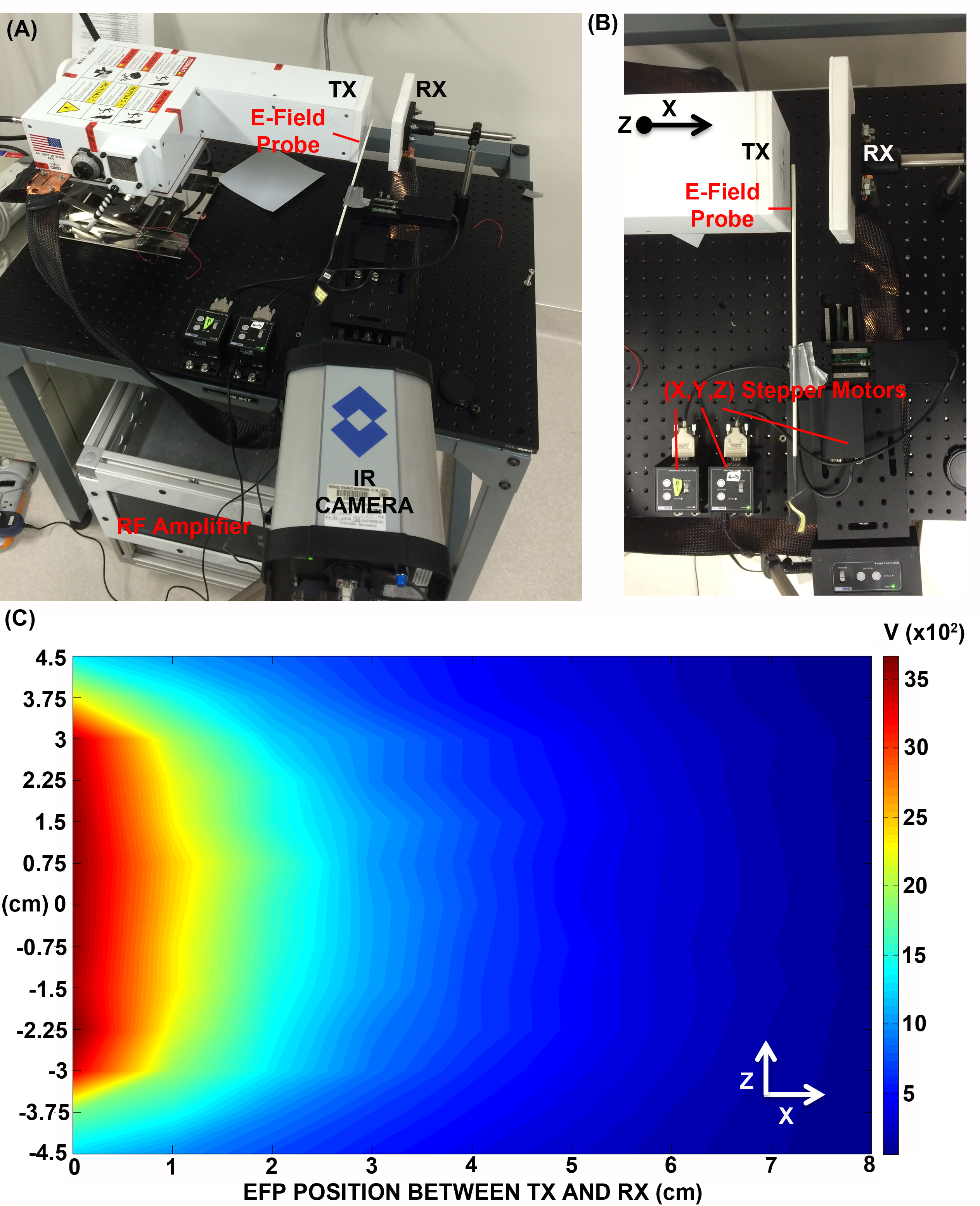
**

**S2. Fig.**  **Setup for extracting electric-field intensities for an RF power of 20 W**.

Supplement: S2 Fig — (A) A custom-made electric field probe (EFP, Thermed Inc, PA, USA) was used to measure voltages in between the transmitting (TX) and receiving (RX) heads of the portable RF-probe (p-RF) via an oscilloscope. The EFP was attached to three stepper motors that could manipulate the probe across the x, y, z-axis. The plane shown in the upper left section of (B) was fully characterized across a TX:RX distance of 8 cm across the full z-axis -4.5 cm to +4.5 cm (NB: the voltages measured lie on the plane perpendicular to the figure). The extracted voltages were then fitted as a contour plot in Matlab and are shown in (C). The direction of the voltage plane is also shown in the bottom right-hand corner. As can be seen, the voltage is concentric around the 0-point and gradually falls off as the distance between the probe and the TX head increases. The voltages were characterized for 20 W, rather than 200 W, as the probe would heat significantly if allowed to go above 40 W and would damage the probe. Also, 8 cm was chosen as this was the TX:RX distance used in all experiments Converting Voltages to Electric fields. The component of electric field in any direction is the negative of rate of change of the potential in that direction. If the differential voltage change is calculated along a direction dx, then it is seen to be equal to the electric field component in that direction times the distance dx. The electric field can then be expressed as E = -dV/dx. Using the voltages extracted above and the distances between each measured voltage (0.01 m) allowed us to calculate the electric-field contour plot shown in the main text (Fig 2D). (DOCX) [file pone.0136382.s002.docx]
